# Association of the systemic immune-inflammation index with all-cause mortality in patients with arteriosclerotic cardiovascular disease

**DOI:** 10.3389/fcvm.2022.952953

**Published:** 2022-09-12

**Authors:** Lu He, Xuegang Xie, Jianying Xue, Hang Xie, Yushun Zhang

**Affiliations:** Department of Structural Heart Disease, The First Affiliated Hospital of Xi’an Jiaotong University, Xi’an, China

**Keywords:** immune-inflammation index, ASCVD, mortality, NHANES, non-linear

## Abstract

**Objective:**

Systemic immune-inflammation index (SII), derived from blood cell counts of circulating platelets, neutrophils, and lymphocytes, has been identified as a novel inflammatory and prognostic marker. However, the clinical value of SII in patients with arteriosclerotic cardiovascular disease (ASCVD) had not been further explored. Thus, this study is designed to explore the associations of SII with mortality in ASCVD individuals.

**Methods:**

All individuals with ASCVD aged ≥20 years were included from the National Health and Nutritional Examination Surveys (NHANES) 2005–2014 and followed for survival until 31 December 2019. Multivariable Cox analysis investigated the associations between SII, evaluated as a continuous variable with splines, as categorical ones (quartiles), and the all-cause death. To demonstrate the association between SII and mortality, subgroup analysis, restricted cubic spline along with piecewise linear regression were also conducted.

**Results:**

A total of 2,595 participants (57.8% men) were included. During a median of 7.7 years of follow-up, 1,122 deaths due to all-cause were recorded. After adjusting for multiple confounders, when compared with the patients in quartile 1 (SII ln transform), those in quartile 4 had a 46% increased risk for all-cause death [hazard ratio (HR) = 1.46, 95% confidence interval (CI) = 1.22–1.75]. As a continuous variable, each unit of raised ln-SII was associated with a 24% increased risk of all-cause death (HR = 1.24, 95% CI = 1.10–1.38). In the restricted cubic spline regression model, the relationship between ln-SII and all-cause death was non-linear. The cutoff value of ln-SII for mortality was 6.57 and those with a higher than the threshold point had a 1.25-fold risk of mortality. No significant difference was noted below the threshold points.

**Conclusion:**

An association was detected between the baseline ln-SII and all-cause mortality in a United States ASCVD population. Increased SII is associated with poor survival in individuals with ASCVD.

## Introduction

Cardiovascular diseases (CVDs) continue to be a significant health burden throughout the world (1). Among the world’s leading causes of morbidity and mortality, atherosclerosis contributes to the majority of cardiovascular events and will remain the leading cause by 2030 (2). The ASCVD caused by atherosclerosis is one of the leading causes of death worldwide. It included non-fatal myocardial infarction (MI), stable or unstable angina, stroke, and coronary heart disease (CHD) (3), which consumes a high proportion of healthcare budgets globally (4). As a result of vascular inflammation, endothelial dysfunction, plaque formation, and diminished oxygen supply to target organs, atherosclerosis is characterized by inflammation of blood vessels and thickening of artery walls (5). Several stages of atherosclerosis are characterized by inflammation, and both innate and adaptive immune systems contribute to its progression that includes the formation and progression of fibro-fatty lesions and final complications (6, 7). According to a recent study, inflammation is responsible for the onset and progression of atherosclerosis (8). Healthy lifestyle patterns can greatly reduce the risk of atherosclerosis by preventing the development of inflammatory changes. Moreover, the Mediterranean diet reduced inflammatory biomarkers and prevented cardiovascular events (9). Atherosclerosis is often considered to be an inflammatory disease with important contributions from the immune system throughout the process (10). During atherosclerosis, inflammation and the immune system play crucial roles in initiation and resolution (11).

Recently, a new index of systemic immune-inflammation (SII), based on platelets, neutrophils, and lymphocytes, has been reported to predict outcomes for patients with multiple cancers and other several different clinical conditions, such as ulcerative colitis, heart failure, acute ischemic stroke, and acute kidney injury (12–16). The SII, calculated by the formula: platelets count × neutrophil/lymphocyte ratio, was developed by Hu et al. in 2014 and has been extensively explored since then (17). Inflammatory indicator SII is a composite indicator integrating neutrophils, lymphocytes, and platelets. They are considered excellent indicators of local immune responses (18). It has been demonstrated that SII is closely related to the poor prognosis of a number of CVDs. ASCVD is known to be accompanied by inflammation, which plays a crucial role in its development and occurrence. However, to our best knowledge, the association between SII and ASCVD prognosis has never been studied, and it is also not known whether a dose-response relationship exists between them. Thus, we will fill the knowledge gap in the ASCVD population of the United States (US).

## Materials and methods

### Study population and design

We analyzed data collected during the 2005–2014 cycles of the National Health and Nutritional Examination Surveys (NHANES), a nationwide cross-sectional study conducted by the National Center for Health Statistics (NCHS) designed to track the overall health and nutrition status of citizens in the United States. To select a representative sample of the United States civilians, a complex multistage probability sampling method was employed for the NHANES. The NCHS approved the protocol, and each participant provided written consent.

**FIGURE 1:**
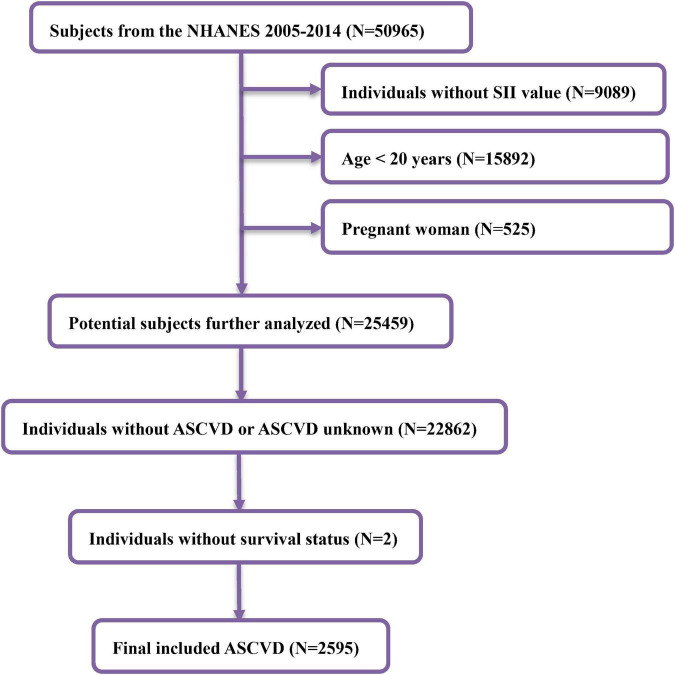
Flowchart diagram of patients’ selection in this study.

In the NHANES 2005–2014, there were a total of 50,965 individuals. We excluded participants without SII value, age <20 years, or pregnant women. Participants without ASCVD at baseline or survival information were excluded. Finally, a total of 2,595 adults were involved in our study. Figure 1 illustrates how to include and exclude participants.

### Definition of systemic immune-inflammation index

A hematology-analyzing device (Coulter^®^DxH 800 Analyzer) was used to determine the lymphocyte, neutrophil, and platelet counts and was expressed as × 10^3^ cells/μl. The SII level was calculated according to previous studies reported as platelet count × neutrophil count/lymphocyte count (17). In our analysis, SII was used as an exposure variable.

### Outcome ascertainment

The NCHS provides links to mortality data used here. The NHANES datasets were linked with the National Death Index (NDI) based on a probabilistic match strategy to obtain data on mortality due to all causes. In the study, the primary outcome was mortality due to all causes, CVD, or cerebrovascular disease until 31 December 2019. As a measure of censoring, we examined the time from enrollment (the date of the interview) to death. These final mortality status, follow-up time, and the underlying leading causes of death files are available for online access.^1^

### Covariates

Covariates used in this study included age (<60 years and ≥60 years), gender (male and female), ethnicity (Mexican American, non-Hispanic Black, non-Hispanic White, and others), family income-to-poverty ratio (PIR), waist circumference (cm), body mass index (BMI) (<18.5, 18.5–24.9, 25–29.9, and ≥30 kg/m^2^), marital status (married/living with partner and widowed/separated/divorced/never married), smoking status (never, former, and now), educational status (less than high school, high school or equivalent, and above high school), alcohol user (never, mild, moderate, and heavy), medical history of asthma, malignancy, stroke, diabetes, and hypertension. The estimated glomerular filtration rate (eGFR) was calculated according to Chronic Kidney Disease Epidemiology Collaboration formula (19). The definition of hypertension was either having a systolic blood pressure (SBP) of 140 or/and a diastolic blood pressure (DBP) of 90 mmHg, taking antihypertensive medications, or having a self-reported history of hypertension. Diabetes was diagnosed based on self-reported diabetes, taking antidiabetic medicines, or hemoglobin A1c (HbA1c) level ≥6.5%. Asthma was diagnosed based on self-reported asthma or use of antiasthmatic drug. The laboratory biochemical parameters were selected *a priori* according to the clinical significance or evidence from the literature. The laboratory indicators used included albumin (g/dl), alanine aminotransferase (ALT; IU/L), aspartate aminotransferase (AST; IU/L), HbA1c (%), total cholesterol (mg/dl), high-density lipoprotein (HDL) cholesterol (mg/dl), serum creatinine (mg/dl), and uric acid (mg/dl). A detailed description of the laboratory testing procedures and quality control strategies was provided at https://www.cdc.gov/nchs/nhanes/.

### Statistical analyses

As part of the NHANES complex sampling design, we utilized appropriate weights to ensure a representative sample of the US national population. Continuous variables were expressed as survey-weighted mean [95% confidence interval (CI)], and categorical variables were presented as a survey-weighted percentage (95% CI). The weighted chi-square test was carried out for the classified variables, and the *p*-value of the continuous variables was calculated with the weighted linear regression model. The SII data, moreover, are unevenly distributed and specifically skewed to the right. Values were ln-transformed before conducting statistical analysis. The ln-SII was grouped into four subgroups by its quartiles, i.e., Q1 (<5.8), Q2 (5.8–6.2), Q3 (6.2–6.6), and Q4 (≥6.6), and Q1 was used as the reference group. To evaluate the linear trend, the median value of each quartile was assigned as a continuous variable. Based on restricted cubic spline plots with three knots, the dose-response relationship between each of the parameters was clarified. The hazard ratios (HRs) and corresponding 95% CI for individuals in Q2, Q3, and Q4 were computed as compared to those in Q1. Additional analysis was undertaken using ln-SII as a continuous variable as well. Three distinct models were constructed using multivariate Cox regression models, i.e., a model without adjustment (no covariates were adjusted), a model with minimal adjustments, and a fully adjusted model. The minimally adjusted model was adjusted for sex, age, BMI, ethnicity, waist circumference, education level, marital status, and family poverty income ratio, and the fully adjusted model was further adjusted for alcohol user, smoke, malignancy, stroke, asthma, diabetes, hypertension, albumin, eGFR, ALT, AST, total cholesterol, HbA1c, HDL cholesterol, creatinine, and uric acid. To examine the threshold effect of the ln-SII on all-cause mortality, we used a two-piecewise linear regression model with a smoothing function. We identified several turning points along a pre-defined interval and then chose the point with the highest model likelihood. The turning point was identified through trial and error that included a selection of inflection points along a pre-defined interval and then picking the inflection points that produce the maximum model likelihood. Furthermore, we tested the log-likelihood ratio of the one-line linear regression model with the two-piecewise linear model. Subgroup analyses were carried out according to patients’ characteristics and comorbidities stratified by age (<60 and ≥60), gender (female and male), marital status (married [married/living with partner] and unmarried [divorced/never married/separated/widowed]), malignancy (yes and no), stroke (yes and no), asthma (yes and no), diabetes (yes and no), hypertension (yes and no), alcohol user (never, mild, moderate, and heavy), smoke (never, former, or now), and eGFR (<60, 60–90, and ≥90). The *p*-values for the product terms between continuous ln-SII and multiple stratification factors were utilized to explore the significance of interactions.

Our results were also subjected to sensitivity analyses to assess their robustness. First, to exclude the possible reverse causality, sensitivity analysis was carried out by removing individuals who died within 2 years of follow-up. Second, a sensitivity analysis was performed among individuals free of a known medical history of malignancy. Furthermore, to account for missing values, a multiple imputation techniques based on 10 replications was used to account for missing values. Then we performed sensitivity analyses using 10 complete datasets, and a pooled result was yielded. Finally, to exclude the effect of drug treatment on the results, we further adjusted for the treatment with the main class of drugs (statins, beta blockers, and antiplatelet). In this study, *p* < 0.05 was considered statistically significant. All analyses were conducted using EpowerStats (X&Y Solutions, Inc., Boston, MA, United States) and R (The R Foundation).

## Results

### Characteristics of arteriosclerotic cardiovascular disease participants

Table 1 exhibits the weighted demographic background characteristics of the included individuals. A total of 2,595 ASCVD were included in the study, of which 57.8% were men, with an average age of 66.7 ± 12.9 years.

**TABLE 1:** Baseline characteristics of ASCVD individuals according to ln transform systemic immune-inflammation index quartiles, weighted.

	Q1 (<5.8)	Q2 (5.8–6.2)	Q3 (6.2–6.6)	Q4 (≥6.6)	*P*-value
Age (years)	64.21 (62.83, 65.59)	63.42 (61.90, 64.94)	65.36 (64.06, 66.65)	66.48 (65.22, 67.75)	0.0242
BMI (kg/m^2^)	29.77 (29.13, 30.40)	29.83 (29.26, 30.39)	30.07 (29.35, 30.79)	30.51 (29.67, 31.34)	0.5297
Waist circumference (cm)	104.41 (103.02, 105.79)	104.74 (103.28, 106.20)	104.93 (103.25, 106.60)	106.69 (104.74, 108.65)	0.2881
Family poverty income ratio	2.65 (2.46, 2.83)	2.50 (2.34, 2.67)	2.58 (2.40, 2.77)	2.43 (2.28, 2.59)	0.3031
Albumin (g/dl)	4.19 (4.15, 4.22)	4.19 (4.16, 4.22)	4.14 (4.11, 4.18)	4.08 (4.03, 4.12)	0.0003
eGFR (ml/min/1.73 m^2^)	75.57 (73.21, 77.92)	77.12 (74.62, 79.61)	73.79 (71.43, 76.14)	69.39 (67.43, 71.35)	< 0.0001
ALT (IU/L)	26.75 (24.96, 28.53)	24.94 (23.02, 26.87)	23.48 (20.96, 25.99)	24.33 (20.15, 28.52)	0.1821
AST (IU/L)	28.72 (27.57, 29.87)	26.64 (25.04, 28.25)	25.29 (24.02, 26.56)	25.53 (23.58, 27.47)	0.0004
Total cholesterol (mg/dl)	182.09 (178.01, 186.18)	183.50 (179.54, 187.46)	182.13 (177.39, 186.87)	184.15 (179.06, 189.25)	0.8596
HbA1c (%)	6.06 (5.97, 6.16)	6.14 (5.99, 6.28)	6.03 (5.92, 6.13)	6.13 (6.01, 6.25)	0.4715
HDL cholesterol (mg/dl)	50.79 (48.85, 52.73)	49.50 (47.98, 51.02)	50.69 (49.39, 51.98)	50.78 (49.20, 52.36)	0.6287
Creatinine (mg/dl)	1.07 (1.03, 1.11)	1.03 (0.98, 1.07)	1.04 (1.01, 1.07)	1.15 (1.09, 1.22)	0.0022
Uric acid (mg/dl)	6.02 (5.88, 6.16)	5.75 (5.59, 5.92)	5.93 (5.79, 6.08)	6.13 (5.98, 6.28)	0.0092
Sex					0.0031
Female	37.87 (32.61, 43.42)	45.33 (40.60, 50.16)	46.22 (41.58, 50.92)	49.80 (45.30, 54.31)	
Male	62.13 (56.58, 67.39)	54.67 (49.84, 59.40)	53.78 (49.08, 58.42)	50.20 (45.69, 54.70)	
Ethnicity					< 0.0001
Mexican American	3.49 (2.46, 4.93)	4.93 (3.51, 6.87)	4.69 (3.02, 7.22)	6.08 (3.98, 9.16)	
Non-Hispanic Black	15.83 (12.51, 19.82)	10.24 (8.17, 12.76)	8.59 (6.91, 10.64)	6.46 (4.94, 8.41)	
Non-Hispanic White	72.51 (67.14, 77.30)	75.96 (72.10, 79.44)	79.66 (75.55, 83.23)	79.05 (74.35, 83.08)	
Other	8.17 (5.99, 11.05)	8.87 (6.50, 12.01)	7.05 (5.12, 9.63)	8.42 (5.99, 11.70)	
Education level					0.2045
Less Than High School	27.04 (22.73, 31.83)	25.34 (21.13, 30.07)	26.83 (22.65, 31.47)	27.01 (22.91, 31.54)	
High school or GED	23.97 (20.49, 27.83)	27.79 (23.54, 32.50)	26.64 (21.81, 32.11)	31.91 (27.61, 36.55)	
Above high school	49.00 (44.01, 54.00)	46.86 (41.75, 52.04)	46.53 (40.27, 52.90)	41.08 (35.84, 46.54)	
Marital status					0.0002
Unmarried	33.48 (28.78, 38.53)	35.21 (30.78, 39.91)	45.08 (40.51, 49.74)	43.42 (38.72, 48.25)	
Married	66.52 (61.47, 71.22)	64.79 (60.09, 69.22)	54.92 (50.26, 59.49)	56.58 (51.75, 61.28)	
Malignancy					0.4415
No	81.23 (76.90, 84.90)	80.95 (77.23, 84.19)	78.78 (74.43, 82.57)	77.29 (72.50, 81.46)	
Yes	18.77 (15.10, 23.10)	19.05 (15.81, 22.77)	21.22 (17.43, 25.57)	22.71 (18.54, 27.50)	
Stroke					0.0721
No	66.24 (61.38, 70.77)	66.94 (61.55, 71.92)	58.61 (53.97, 63.10)	62.92 (57.77, 67.79)	
Yes	33.76 (29.23, 38.62)	33.06 (28.08, 38.45)	41.39 (36.90, 46.03)	37.08 (32.21, 42.23)	
Asthma					0.0268
No	80.31 (75.60, 84.31)	85.08 (80.09, 88.99)	80.00 (75.46, 83.89)	75.98 (72.02, 79.54)	
Yes	19.69 (15.69, 24.40)	14.92 (11.01, 19.91)	20.00 (16.11, 24.54)	24.02 (20.46, 27.98)	
Diabetes					0.3251
No	69.36 (65.37, 73.08)	67.29 (62.52, 71.73)	69.51 (64.95, 73.71)	64.53 (59.97, 68.84)	
Yes	30.64 (26.92, 34.63)	32.71 (28.27, 37.48)	30.49 (26.29, 35.05)	35.47 (31.16, 40.03)	
Alcohol user					0.6014
Never	46.35 (41.48, 51.29)	50.72 (44.93, 56.49)	47.28 (41.99, 52.64)	52.65 (48.51, 56.75)	
Mild	36.67 (31.61, 42.05)	30.80 (26.05, 36.00)	36.12 (31.31, 41.23)	29.88 (25.32, 34.87)	
Moderate	7.40 (4.65, 11.58)	7.82 (5.11, 11.77)	7.35 (5.16, 10.39)	9.08 (7.09, 11.57)	
Heavy	9.58 (7.06, 12.87)	10.66 (7.46, 15.02)	9.24 (6.83, 12.39)	8.39 (5.63, 12.33)	
Smoke					0.1728
Never	40.28 (36.20, 44.50)	41.96 (37.09, 46.98)	36.54 (32.00, 41.33)	35.73 (31.26, 40.46)	
Former	38.92 (34.32, 43.74)	36.30 (32.27, 40.54)	43.07 (37.85, 48.44)	38.44 (33.93, 43.16)	
Now	20.80 (17.11, 25.04)	21.74 (18.20, 25.76)	20.39 (16.44, 25.01)	25.83 (21.88, 30.21)	
Hypertension					0.0617
No	24.85 (20.29, 30.04)	30.51 (25.62, 35.90)	25.15 (20.70, 30.20)	21.38 (17.66, 25.63)	
Yes	75.15 (69.96, 79.71)	69.49 (64.10, 74.38)	74.85 (69.80, 79.30)	78.62 (74.37, 82.34)	

Mean for continuous variables: the p-value was calculated by the weighted linear regression. Percent for categorical variables: p-value was calculated by weighted chi-square test.

Overall, there were significant differences in age, albumin, eGFR, AST, creatinine, uric acid, sex, ethnicity, marital status, and history of asthma among ln transform SII quartiles (all *p* for trend <0.05).

### Systemic immune-inflammation index and the risk of all−cause mortality

There were a total of 1,122 deaths detected during a median follow-up of 92.6 months (interquartile range, 2–179 months). The association between ln-SII and all-cause mortality in patients with ASCVD is shown in Table 2. Compared with participants in Q1, the multivariate minimally and fully adjusted HRs (95% CI) for those in Q4 were 1.49 (1.23–1.81) and 1.46 (1.22–1.75), respectively, for all-cause mortality (all *p* trend < 0.001). When the ln-SII was treated as a continuous variable, we found that for per 1-unit increment in ln-SII, there was a 27% higher risk of all-cause mortality in the minimally adjusted model (HR = 1.27, 95% CI = 1.12–1.43; *p* = 0.0002) and 24% in the fully adjusted model (HR = 1.24, 95% CI = 1.10–1.38; *p* = 0.0003).

**TABLE 2:** Associations of the SII ln transform with all-cause mortality in individuals with ASCVD.

	Non-adjusted	Adjust I	Adjust II
SII ln transform	1.42 (1.28, 1.57) < 0.0001	1.27 (1.12, 1.43) 0.0002	1.24 (1.10, 1.38) 0.0003
**SII ln transform quartiles**			
Q1	1 (Reference)	1 (Reference)	1 (Reference)
Q2	1.02 (0.86, 1.22) 0.7931	1.00 (0.81, 1.22) 0.9707	1.05 (0.87, 1.27) 0.6206
Q3	1.11 (0.93, 1.32) 0.2479	1.00 (0.82, 1.22) 0.9861	1.06 (0.87, 1.28) 0.5703
Q4	1.76 (1.50, 2.08) < 0.0001	1.49 (1.23, 1.81) < 0.0001	1.46 (1.22, 1.75) < 0.0001
*P* for trend	<0.001	<0.001	<0.001

Non-adjusted model adjusts for: None.Adjust I model adjust for: sex, age, BMI, ethnicity, waist circumference, education level, marital status, and family poverty income ratio.Adjust II model adjust for: sex, age, BMI, ethnicity, waist circumference, education level, marital status, family poverty income ratio, alcohol user, smoke, malignancy, stroke, asthma, diabetes, hypertension, albumin, eGFR, ALT, AST, total cholesterol, HbA1c, HDL cholesterol, creatinine, and uric acid.

### The analyses of a non-linear relationship

**FIGURE 2:**
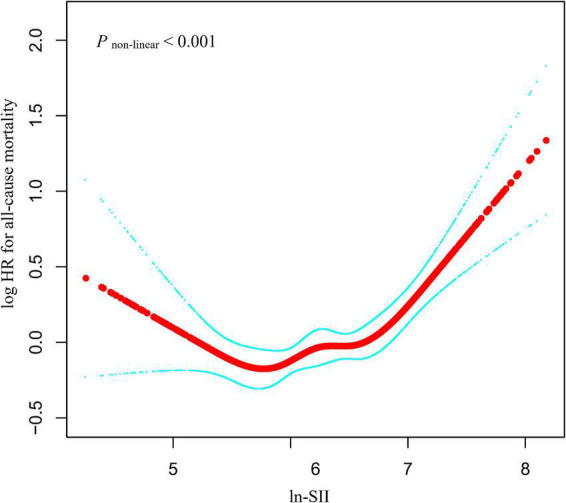
Relationship between ln-SII and all-cause mortality by smooth curve fitting. Adjustment for sex, age, body mass index (BMI), ethnicity, waist circumference, education level, marital status, family poverty income ratio, alcohol user, smoke, malignancy, stroke, asthma, diabetes, hypertension, albumin, estimated glomerular filtration rate (eGFR), alanine aminotransferase (ALT), aspartate aminotransferase (AST), total cholesterol, hemoglobin A1c (HbA1c), high-density lipoprotein (HDL) cholesterol, creatinine, and uric acid. The red line demonstrates the risk of mortality, and the blue dotted lines illustrate its 95% confidence interval (CI).

Using smooth curve fitting, a non-linear relationship between ln-SII and all-cause death was discovered after adjusting for multiple potential confounders (Figure 2; *p* < 0.001). Moreover, the two-piecewise linear regression demonstrated that the possibility of all-cause death gradually reduced up to a minimum at the ln-SII value of 6.57 (SII = 713.4 × 10^9^) and then increased with higher ln-SII values. Below the threshold, per one unit increment in ln-SII was not associated with the risk of all-cause death [adjusted hazard ratio (aHR) = 0.99, 95% CI: 0.83–1.19, *p* = 0.946]. Nevertheless, above the threshold, ln-SII was associated with a higher risk of all-cause death (aHR = 2.25, 95% CI: 1.68–3.02, *p* < 0.001; Table 3). Based on the log-likelihood ratio test, the model differences were statistically significant (*p* < 0.001).

**TABLE 3:** Threshold analysis for the relationship between ln-SII and all-cause mortality in patients with ASCVD.

Models	Adjusted HR (95%CI) *P*-value
**Model I**	
One line slope	1.30 (1.14, 1.48) < 0.0001
**Model II**	
Turning point (K)	6.57
<6.57	0.99 (0.83, 1.19) 0.9460
>6.57	2.25 (1.68, 3.02) < 0.0001
HR between <6.57 and >6.57	2.27 (1.51, 3.40) < 0.0001
Logarithmic likelihood ratio test	<0.001

Adjust for: sex, age, BMI, ethnicity, waist circumference, education level, marital status, family poverty income ratio, alcohol user, smoke, malignancy, stroke, asthma, diabetes, hypertension, albumin, eGFR, ALT, AST, total cholesterol, HbA1c, HDL cholesterol, creatinine, and uric acid.

### Subgroup analyses and sensitivity analyses

**FIGURE 3:**
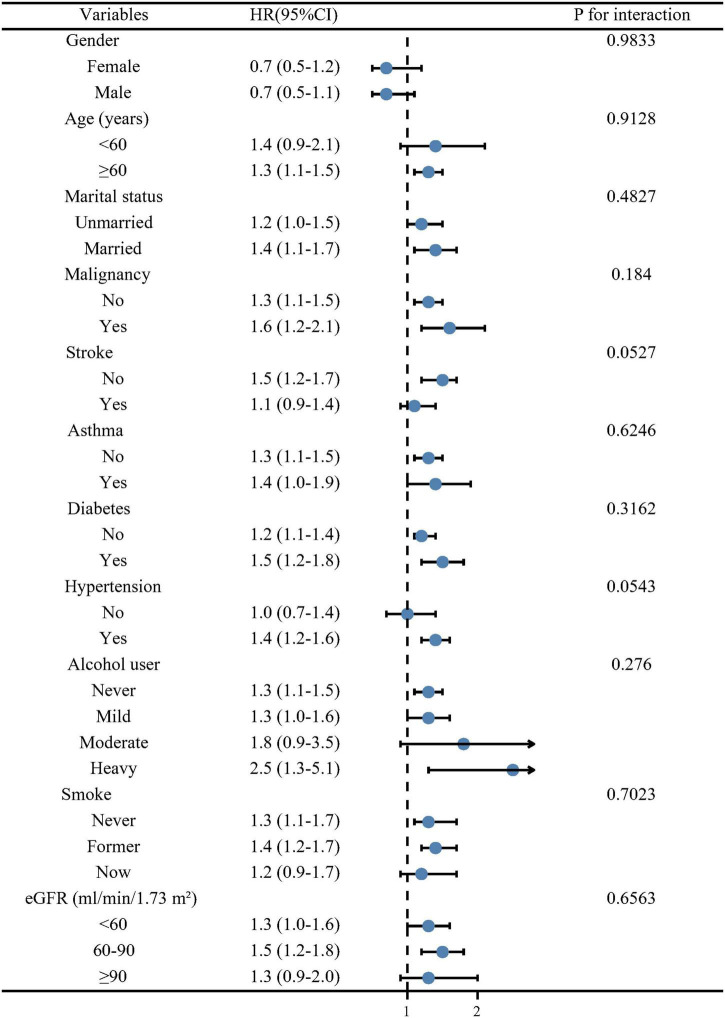
Forest plot of subgroup analysis for all-cause mortality. Hazard ratios (HRs) were calculated using multivariate Cox regression models adjusted for the variables listed in the fully adjusted model except for the variable used for stratification.

In order to evaluate whether patient characteristics and comorbidities could account for the association between ln-SII and all-cause mortality, we calculated an adjusted multivariable HR for all-cause mortality stratified by age (<60 and ≥60 years), gender (female and male), malignancy (yes and no), stroke (yes and no), asthma (yes and no), marital status (married and unmarried), diabetes (yes and no), hypertension (yes and no), alcohol user (never, mild, moderate, and heavy), smoke (never, former, and now), and eGFR (<60, 60–90, and ≥90 ml/min/1.73 m^2^). We observed the robust association between ln-SII and long-term death in most subgroups (Figure 3). There was no significant interaction with these stratification variables on the association of ln-SII with all-cause death (all *p* > 0.05).

**FIGURE 4:**
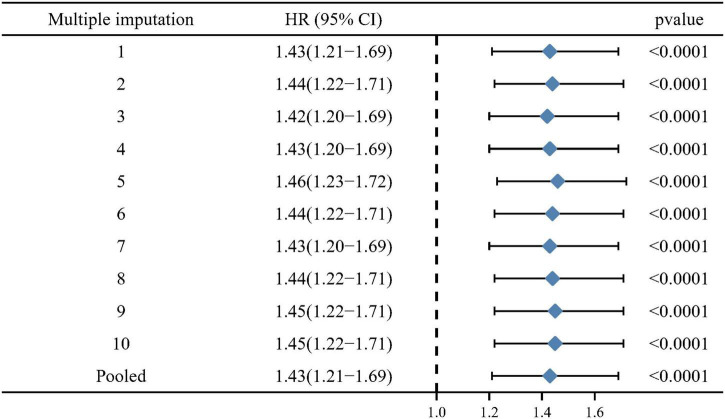
Pooled results for multiple imputations (10 times) of missing data.

For the purposes of minimizing the potential for reverse causality, deaths within the first 2 years of follow-up were excluded. When Q1 was used as a reference, the sensitivity analysis presented the similar results with statistical significance, as well as in unadjusted model (HR = 1.62, 95% CI: 1.35–1.94, *p* < 0.0001), minimally adjusted model (HR = 1.37, 95% CI: 1.11–1.68, *p* = 0.0035), and fully adjusted model (HR = 1.34, 95% CI: 1.08–1.66, *p* = 0.0085). The results persisted when ln-SII was treated as continuous variables (Supplementary Table 1). Furthermore, when individuals with history of malignancy were excluded, the main results still remained stable in unadjusted model (HR = 1.7, 95% CI: 1.4–2.0, *p* < 0.001), minimally adjusted model (HR = 1.5, 95% CI: 1.2–1.8, *p* < 0.001), and fully adjusted model (HR = 1.5, 95% CI: 1.2–1.9, *p* < 0.001; Supplementary Table 2). Moreover, multiple imputations (10 times) were performed to ensure that missing data would not influence the results. A multiple imputation sensitivity analysis was conducted but did not significantly alter the results (HR = 1.43, 95% CI: 1.21–1.69, *p* < 0.001; Figure 4). When further adjusted for the treatment with the main class of drugs (statins, beta blockers, and antiplatelet) in model II, the main results persisted (Supplementary Table 3).

## Discussion

Globally, ASCVD continues to be the leading cause of death. In ASCVD, immune and inflammatory responses are demonstrated to play a key role in all stages of vascular lesions formation (8). It is well documented that inflammatory markers, such as neutrophils, lymphocytes, monocytes, and platelets, play a critical role in coronary atherosclerosis (20, 21). Thus, the SII combines the three major immune cells and has received considerable attention in CVD. For example, studies have demonstrated that SII is a better predictor of mortality in individuals performing transcatheter aortic valve implant for severe aortic stenosis and coronary artery disease than the neutrophil/lymphocyte ratio and platelet/lymphocyte ratio (22). Thus, the SII should be explored in ASCVD.

To our knowledge, this is the first study designed for investigating the association between SII and the risk of all-cause death in ASCVD in a large representative sample of the US adults. There have been some studies showing associations between individual leukocyte subtypes and cardiovascular events, but our study is uniquely positioned to test the association between all of them simultaneously. The main results demonstrated that higher ln-SII was strongly associated with all-cause mortality in ASCVD, independent of multiple confounding variables. These results were observed in both continuous and categorical ln-SII analyses. A non-linear curve for the association with all-cause death was found in the ASCVD population with a threshold value of 6.57 of ln-SII. Below the threshold, every one-unit increment in ln-SII was not associated with the risk of all-cause death. Nevertheless, above the threshold, ln-SII was associated with a higher probability of mortality. The robustness of the results was demonstrated by stratified analyses and sensitivity analyses.

As a new inflammatory biomarker, SII could assess the immune and inflammatory responses in a more balanced and comprehensive manner (23). As SII levels increase, inflammatory activity in various diseases might be high, leading to poor clinical outcomes (24). Having high SII values has been demonstrated to be a powerful tool in predicting survival outcomes in cancer, chronic patients with heart failure, and long-term survival in elderly non-ST-elevation patients with MI (12, 25, 26). The platelets play a critical role in prothrombotic potential during arterial thrombosis, as well as in atherogenesis and inflammation. Platelets have a central role in maintaining hemostasis and coagulation and prevention of hemorrhage in newly formed tumor vasculature (27, 28). Platelets are actively involved in the inflammatory and atherosclerotic process through interfacing with the endothelium, leukocytes, and non-activated platelets, and they promote the progression of atherosclerosis (29, 30). In the blood, neutrophils are the most abundant subtype of white blood cells and play a vital role in mediating inflammation. It has been demonstrated that neutrophils result in tissue damage and inflammation in advanced stages of atherosclerosis by causing smooth muscle cells to lyse and die (31). Furthermore, studies have demonstrated that neutrophils affect key biological processes related to atherosclerosis, thrombosis, and ischemic stroke by interacting with platelets (32). In every phase of the atherosclerotic process, lymphocytes play a vital role in regulating the inflammatory response. Low lymphocyte counts are associated with the progression of atherosclerosis. As the lipid core of an atherosclerotic plaque develops, it ruptures and forms a thrombus after lymphocytes undergo apoptosis (33). Furthermore, it has been confirmed that a low lymphocyte count is associated with cardiovascular events in a positive manner and associated with a worse prognosis in numerous diseases, such as stable coronary artery disease (34). Thus, in the light of the abovementioned evidence, it is reasonable to demonstrate a non-linear relationship between ln-SII and mortality in a US ASCVD population. SII is a simple, inexpensive inflammatory biomarker that can be obtained from complete blood work. Thus, SII has a great potential for clinical application in ASCVD.

Our study has some strengths and limitations. Most importantly, the study was based on a nationally representative large sample size cohort with the chance to adjust for a comprehensive range of confounders and the use of an NDI death record. Thus, it is possible to extrapolate the findings of this study to the general adult population in the United States. The study’s prospective design offers another strength with ASCVD individuals being followed up for an average of 7.7 years, and follow-up periods were long enough for a sufficient number of deaths to be observed. A third strength is that multiple different sensitivity analyses were conducted to test the robustness of our results. Nevertheless, this study has certain limitations. First, due to the actual limitations of NHANES’ own design, platelet, neutrophil, and lymphocyte count were measured at a single time point at baseline, which may have changed over time during the follow-up. Second, there was no information about how ASCVD and other cardiovascular risk factors were treated following the baseline interview in this study. Third, despite adjusting for many potential confounding factors, we could not completely rule out the effects of unmeasured confounders.

## Conclusion

Using a large nationally representative survey in individuals among the US adults, the study revealed that higher SII was non-linear and associated with poor survival in ASCVD with a threshold value of 6.57 of ln-SII. Below the threshold, every one-unit increment in ln-SII was not associated with the possibility of all-cause death. Nevertheless, above the threshold, ln-SII was associated with a higher probability of all-cause death. Further studies will be needed to demonstrate whether lowering SII will improve the survival in ASCVD.

## Data availability statement

Publicly available datasets were analyzed in this study. This data can be found here: The National Health and Nutrition Examination Survey dataset at https://www.cdc.gov/nchs/nhanes/index.htm.

## Ethics statement

This study was reviewed and approved by the NCHS. Written informed consent was obtained from all participants for their participation in this study.

## Author contributions

LH and YZ created the study protocol. LH, XX, and HX contributed to the analysis plan. LH, XX, JX, and HX wrote the first draft of the manuscript. YZ revised the manuscript to create the final version. All authors contributed to the design of the study protocol and reviewed the manuscript.

## Footnotes

1
https://ftp.cdc.gov/pub/Health_Statistics/NCHS/datalinkage/linked_mortality/
