# Supplementary material for: Association of the systemic immune-inflammation index with all-cause mortality in patients with arteriosclerotic cardiovascular disease
Source: Front Cardiovasc Med. 2022 Sep 12;9:952953. doi: 10.3389/fcvm.2022.952953 (PMC9510918; doi:10.3389/fcvm.2022.952953)
Supplement: Supplementary file 1 [file Data_Sheet_1.pdf]

Table S1 Sensitivity analyses after excluding patients who died within 2 years of follow up

	Non-adjusted	Adjust I	Adjust II
SII ln transform	1.31 (1.16, 1.47) <0.0001	1.18 (1.03, 1.35) 0.0176	1.15 (1.00, 1.33) 0.0453
SII ln transform quartiles			
Q1	1.0(Reference)	1.0(Reference)	1.0(Reference)
Q2	1.08 (0.90, 1.31) 0.4065	1.04 (0.84, 1.29) 0.7443	1.08 (0.87, 1.35) 0.4930
Q3	1.16 (0.96, 1.40) 0.1349	1.01 (0.82, 1.26) 0.8946	1.06 (0.85, 1.32) 0.6292
Q4	1.62 (1.35, 1.94) <0.0001	1.37 (1.11, 1.68) 0.0035	1.34 (1.08, 1.66) 0.0085
P for trend	< 0.001	0.019	0.038

Non-adjusted model adjust for: None
Adjust I model adjust for: sex, age, BMI, ethnicity, waist circumference, education level, marital status, and family poverty income ratio;
Adjust II model adjust for: sex, age, BMI, ethnicity, waist circumference, education level, marital status, family poverty income ratio, alcohol user, smoke, malignancy, stroke, asthma, diabetes, hypertension, albumin, eGFR, ALT, AST, total cholesterol, HbA1c, HDL cholesterol, creatinine, and uric acid.

Table S2 Sensitivity analyses after excluding patients with any cancers at baseline

	Non-adjusted	Adjust I	Adjust II
SII ln transform	1.4 (1.2, 1.6) <0.001	1.2 (1.1, 1.4) 0.005	1.2 (1.0, 1.4) 0.014
SII ln transform quartiles			
Q1	1.0	1.0	1.0
Q2	1.0 (0.8, 1.2) 0.912	1.0 (0.8, 1.3) 0.945	1.1 (0.9, 1.4) 0.413
Q3	1.1 (0.9, 1.3) 0.373	1.0 (0.8, 1.2) 0.752	1.0 (0.8, 1.3) 0.819
Q4	1.7 (1.4, 2.0) < 0.001	1.5 (1.2, 1.8) < 0.001	1.5 (1.2, 1.9) < 0.001
P for trend	< 0.001	< 0.001	< 0.001

Non-adjusted model adjust for: None
Adjust I model adjust for: sex, age, BMI, ethnicity, waist circumference, education level, marital status, and family poverty income ratio;
Adjust II model adjust for: sex, age, BMI, ethnicity, waist circumference, education level, marital status, family poverty income ratio, alcohol user, smoke, stroke, asthma, diabetes, hypertension, albumin, eGFR, ALT, AST, total cholesterol, HbA1c, HDL cholesterol, creatinine, and uric acid.

Table S3 Sensitivity analyses further adjust for main class of drugs (statins, beta blockers, antiplatelet) in individuals with ASCVD

	Non-adjusted	Adjust I	Adjust II
SII ln transform	1.42 (1.28, 1.57) <0.0001	1.27 (1.12, 1.43) 0.0002	1.23 (1.10, 1.38) 0.0003
SII ln transform quartiles			
Q1	1.0(Reference)	1.0(Reference)	1.0(Reference)
Q2	1.02 (0.86, 1.22) 0.7931	1.00 (0.81, 1.22) 0.9707	1.05 (0.86, 1.27) 0.6515
Q3	1.11 (0.93, 1.32) 0.2479	1.00 (0.82, 1.22) 0.9861	1.05 (0.86, 1.27) 0.6392
Q4	1.76 (1.50, 2.08) <0.0001	1.49 (1.23, 1.81) <0.0001	1.45 (1.21, 1.74) <0.0001
P for trend	<0.001	<0.001	<0.001

Non-adjusted model adjust for: None
Adjust I model adjust for: sex, age, BMI, ethnicity, waist circumference, education level, marital status, and family poverty income ratio;
Adjust II model adjust for: sex, age, BMI, ethnicity, waist circumference, education level, marital status, family poverty income ratio, alcohol user, smoke, malignancy, stroke, asthma, diabetes, hypertension, albumin, eGFR, ALT, AST, total cholesterol, HbA1c, HDL cholesterol, creatinine, uric acid, and main class of drugs (statins, beta blockers, antiplatelet).
